# Pesticide exposure and risk of Parkinson's disease: A family-based case-control study

**DOI:** 10.1186/1471-2377-8-6

**Published:** 2008-03-28

**Authors:** Dana B Hancock, Eden R Martin, Gregory M Mayhew, Jeffrey M Stajich, Rita Jewett, Mark A Stacy, Burton L Scott, Jeffery M Vance, William K Scott

**Affiliations:** 1 Center for Human Genetics, Duke University Medical Center, Durham, NC, USA; 2 Department of Medicine, Duke University Medical Center, Durham, NC, USA; 3 Miami Institute for Human Genomics, University of Miami Miller School of Medicine, Miami, FL, USA

## Abstract

**Background:**

Pesticides and correlated lifestyle factors (e.g., exposure to well-water and farming) are repeatedly reported risk factors for Parkinson's disease (PD), but few family-based studies have examined these relationships.

**Methods:**

Using 319 cases and 296 relative and other controls, associations of direct pesticide application, well-water consumption, and farming residences/occupations with PD were examined using generalized estimating equations while controlling for age-at-examination, sex, cigarette smoking, and caffeine consumption.

**Results:**

Overall, individuals with PD were significantly more likely to report direct pesticide application than their unaffected relatives (odds ratio = 1.61; 95% confidence interval, 1.13–2.29). Frequency, duration, and cumulative exposure were also significantly associated with PD in a dose-response pattern (*p *≤ 0.013). Associations of direct pesticide application did not vary by sex but were modified by family history of PD, as significant associations were restricted to individuals with no family history. When classifying pesticides by functional type, both insecticides and herbicides were found to significantly increase risk of PD. Two specific insecticide classes, organochlorines and organophosphorus compounds, were significantly associated with PD. Consuming well-water and living/working on a farm were not associated with PD.

**Conclusion:**

These data corroborate positive associations of broadly defined pesticide exposure with PD in families, particularly for sporadic PD. These data also implicate a few specific classes of pesticides in PD and thus emphasize the need to consider a more narrow definition of pesticides in future studies.

## Background

Parkinson's disease (PD) is characterized by progressive depletion of dopaminergic neurons in the substantia nigra that manifests clinically as resting tremor, rigidity, and bradykinesia. Causal genetic variants in multiple genes (*parkin*, *α-synuclein*, *DJ-1*, *PINK1*, and *LRRK2*) have been identified, but altogether, these rare genetic risk factors account for a small fraction of the overall prevalence of PD [1]. The remaining majority of PD cases are likely due to genetic susceptibility variants, environmental influences, and complex gene-environment interactions.

Several epidemiologic studies have supported the hypothesis that broadly defined pesticide exposure may increase risk of PD [2-7], but not all studies examining this relationship have reported a significant positive association [8-10]. Despite the inconclusive data, meta-analysis of case-control studies conducted in the United States suggested that individuals with PD are over two times as likely to report ever being exposed to pesticides as compared to unaffected individuals [11]. Few studies have assessed associations between specific classes of pesticides and PD. Further, farming residence and well-water consumption are considered proxies for pesticide exposure, but meta-analysis suggested weaker associations between these factors and PD [11].

Most studies examining the relationship between pesticide exposure and PD have been conducted in samples of unrelated individuals. Two previous family-based studies examined this relationship in affected sibling pairs [9,12], but the association of pesticide exposure with PD has never been reported in a data set of cases and their unaffected relatives, who are generally well-matched on unmeasured genetic and environmental factors that may predispose to exposure behaviors and disease. When examining environmental associations in such family-based samples, there is less likelihood for spurious association due to such influences as compared to population-based samples, although this advantage is offset by reduced power due to potential overmatching on environmental risk factors. The aim of this family-based case-control study was to examine the associations of self-reported direct pesticide application defined broadly and specifically, farming residences and occupations, and well-water consumption with PD.

## Methods

### Family-based ascertainment

Individuals in our family-based case-control study were recruited from 2000 to 2006 by the Morris K. Udall PD Research Center of Excellence at Duke University Medical Center to identify genetic and environmental factors that influence the risk of PD. Probands were recruited from Duke University Medical Center clinics, other physician referrals, and self referrals. Individuals from referring physicians were introduced to the study by posted study information or direct physician contact, while self referring individuals were introduced to the study through presentations by study staff at local support groups, the Udall Center web site, other individuals, or media coverage of Udall Center activities. Upon enrollment, probands were asked to contact their affected and unaffected relatives and to request their participation. In families with only one individual with PD, siblings, parents, and spouses were recruited. In families with multiple individuals with PD, all relatives with PD were recruited, and the siblings, parents, and spouses of all individuals with PD were recruited along with relatives connecting the branches of the family. Relatives who agreed to participate were contacted by study staff for enrollment into the study. The difficulty of matching referred cases to appropriate controls was addressed with the family-based nature of the study, as cases and relative controls were well matched on genetic and demographic factors and were thus taken from the same underlying population. However, the participation rates among cases and controls could not be determined. The referral-based nature of the study prevented a sampling frame from being established for cases. For family-based controls, no restriction was placed on the number collected, but other factors, including geography, willingness to participate, enrollment cost, and age, were used to prioritize enrollment of controls when multiple existed. Therefore, there is no simple way to determine the denominator for calculating a participation rate in controls.

All study protocols were approved by the Institutional Review Board at Duke University, and approved consent forms were signed by all individuals prior to enrollment. The enrollment procedure involved a blood sample collection to use as a DNA source for our genetic studies, a detailed medical history questionnaire, at least a three-generation family history report, a standard cognitive status test (either the Blessed Orientation-Memory Concentration test or the Modified Mini-Mental State examination), and an environmental risk factor questionnaire.

The structured, 30 to 45-minute telephone environmental risk factor questionnaire was administered by trained interviewers to gather detailed environmental risk factor data on demographics, health habits, and pesticide and other chemical exposures (see Additional file 1: Risk factor questions used to assess residences, occupations, and pesticide applications). The initial questionnaire was evaluated for face validity by several researchers in the PD research field. Small-scale pilot testing was then performed, and the questionnaire was accordingly revised for procedural content. The final questionnaire implemented in this study has not been formally evaluated for reliability over time.

All individuals were given a standard in-person clinical examination utilizing the full Unified PD Rating Scale (UPDRS) to determine affection status and symptom severity. Individuals with PD demonstrated at least two cardinal signs of PD (resting tremor, rigidity, and/or bradykinesia), an asymmetry of symptom onset, and no atypical signs during examination by a board-certified neurologist. Individuals with PD self-reported age-at-onset, defined as the age at which the first cardinal sign was noticed. A board-certified neurologist, physician assistant, or registered nurse examined unaffected individuals, who had no signs of PD, and unclear individuals, who had only one cardinal sign, a history of encephalitis, neuroleptic therapy within a year prior to diagnosis, evidence of normal pressure hydrocephalus, or unusual clinical features suggestive of atypical or secondary parkinsonism. Individuals with an unclear diagnosis were referred to movement disorder specialists for further examination and excluded from our analyses to minimize phenotypic misclassification. Families with more than one individual with PD, as identified by clinical examination for sampled members or by family history report for members not sampled, were considered positive history families. Otherwise, families with only one individual with PD were considered negative history families.

### Measures of pesticide exposure

In the environmental risk factor telephone questionnaire, direct pesticide application was assessed with the following question: "Have you ever applied pesticides to kill weeds, insects, or fungus at work, in your home, in your garden, or on your lawn?" Individuals provided only a "yes/no" answer for this question, so separation of residential applications from occupational applications for analysis was not possible. If the answer was "yes", individuals were asked to list the name of any pesticides they remembered using. For each pesticide, individuals were asked the number of days it was used per year, whether it was currently being used, the years application started and stopped (if applicable), and whether protective gear, such as a mask, rubber gloves, or rubber boots, was used during application. Application of any pesticide chemical by spreading solid granules, spraying by hand, spraying by tractor, spraying by airplane, putting in irrigation water, or placing pest strips or traps was considered a direct pesticide application. Those who reported a direct application of any pesticide that was initiated prior to the reference age were classified as ever exposed. Otherwise, individuals were classified as never exposed. For cases, reference age equated to age-at-onset, and for controls, reference age equated to age-at-examination (AAE) minus the mean disease duration among cases. This adjustment was necessary to give cases and controls comparable exposure periods. For ever exposed individuals, frequency (days per year) and duration (years prior to reference age) were summed across all reported direct pesticide applications, and cumulative exposure was calculated as the multiplicative result of frequency and duration. Frequency, duration, and cumulative exposure were divided into tertiles of exposure, such that the high exposure category included values greater than the upper tertile value (value that 67% of the data is equal to or less than), the middle exposure category included values between the upper tertile value and the lower tertile value (value that 33% of the data is equal to or less than), and the low exposure category included values less than the lower tertile value. The referent level of never being exposed was used as a fourth category.

In order to move beyond a broad assessment of direct pesticide application, the recalled pesticide products were classified into specific functional types (e.g., insecticides, herbicides, and fungicides) and chemical classes. The chemical or trade name of each pesticide product was input into the Pesticide Action Network pesticides database [13] to obtain its primary function (herbicide, insecticide, fungicide, or multiple uses) and the chemical class of the main active ingredient. This database has been used for classification in other pesticide studies [14,15]. For each functional type and chemical class, individuals were classified as ever exposed to the relevant class or type, ever exposed to any other pesticide, and never exposed to any pesticide.

In the residential history section of the environmental risk factor questionnaire, individuals were asked to "list the cities, towns, or communities in which you have lived for the majority of each year from childhood to now." For each location, individuals were then questioned on their years of residence, whether they lived on or next to a farm, whether they drank well-water, and if so, the years of drinking well-water. Individuals who reported drinking well-water at any residence prior to their reference age were classified as ever exposed to well-water consumption. Cumulative duration (years prior to reference age) was then determined and categorized into tertiles of exposure (high, middle, and low exposure categories) and a referent level of never being exposed.

In the occupational history section of the questionnaire, individuals were asked to list their full or part-time jobs held for a year or longer and the years worked at each job. Individuals were asked each job title and company name, from which study staff matched the appropriate Standard Occupational Classification job code of the United States Department of Labor Bureau of Labor Statistics [16]. The following job categories (and job codes) were considered farming occupations: farm, ranch, and other agricultural managers (11–9011); farmers/ranchers (11–9012); supervisors/managers of farming, fishing, and forestry workers (45–1011); farm labor contractors (45–1012); agricultural inspectors (45–2011); animal breeders (45–2021); graders/sorters, agricultural products (45–2091); farmworkers/laborers, crop, nursery, and greenhouse (45–2092); farmworkers, farm and ranch animals (45–2093); agricultural workers, all other (45–2099). Individuals whose residence report revealed that they lived on or next to a farm prior to their reference age or occupation report revealed that they worked in a farming occupation prior to their reference age were classified as ever exposed to farming. Cumulative duration (years of residential or occupational exposure to farming prior to reference age) was calculated and categorized into tertiles of exposure (high, middle, and low exposure categories) and a referent level of never being exposed. History (ever vs. never) and cumulative duration of farming residences and occupations, separately, were also examined.

### Statistical analysis

Even though family-based case-control data sets are robust to within-family confounding by ethnicity, bias due to across-family confounding by ethnicity may still exist. To minimize this bias, we stratified families by self-reported race/ethnicity. Only the white families (308 of 322 ascertained families) provided sufficient statistical power, so analyses were performed in this subset only.

Population-averaged generalized estimating equations (GEE) as implemented by SAS version 8e (SAS Institute, Cary, NC) were used to model the associations between pesticide exposure and PD. GEE modeling requires specification of a within-cluster correlation matrix structure, which serves as the basis for deriving the working correlation matrix from the data. Correlations from this working correlation matrix are then used as nuisance parameters to estimate the across-population regression parameters and corresponding robust variance estimates [17]. GEE with an independence correlation matrix, which begins with the assumption of no correlation between relatives but later estimates correlations to appropriately adjust the variance of association tests, is a valid test for association of an environmental risk factor using pedigrees of similar size and structure to the current data set [18]. The independence matrix was specified in our GEE models, but even if this correlation matrix does not accurately fit the data, GEE models are generally robust to misspecification of the correlation matrix [19].

GEE models using affection status as the outcome assessed associations of history, frequency, duration, and cumulative exposure for broadly defined direct pesticide application, associations of history for direct pesticide application as divided into pesticide functional types and chemical classes, and associations of history and duration for well-water consumption and farming residences/occupations. Individuals never exposed to the relevant measure served as the referent group. Sex and AAE were included in the models as confounders given significant differences between cases and controls. Cigarette smoking and caffeine (coffee, tea, or soft drink) consumption histories (1 = ever, 0 = never) were also included as confounders given significant inverse associations between these environmental factors and PD in this data set [20].

Two types of GEE models were constructed. GEE model 1 tested for the trend of effects across the exposure categories with an ordinal variable (3 = high, 2 = moderate, 1 = low, 0 = never) for frequency, duration, and cumulative exposure. GEE model 2 tested the effect of each exposure category with indicator variables (ever versus never or high, moderate, and low versus never) for each measure (history, frequency, duration, and cumulative exposure). Two-sided *p*-values are presented for model 1 to show the significance of the linear trend tests, while the adjusted odds ratios (ORs) and 95% confidence interval (CI) are presented for model 2 to show the strength of association for each exposure level. Associations of direct pesticide application, well-water consumption, and farming residences and occupations with PD were evaluated in the overall data and in the data stratified by sex and by family history. These analyses were repeated with only adulthood (≥ 18 years old) exposures considered. Associations of direct pesticide application as divided into functional types and chemical classes were only evaluated in the overall data to maintain sufficient statistical power.

### Statistical power

Data sets resembling the actual PD sample were created using the simulation of linkage and association program [21]. Given that the actual PD sample consists mostly of sibling pairs with at least one affected individual, the simulated data sets were created with 300 such sibling pairs to resemble the overall sample, 200 sibling pairs to resemble the negative family history stratum, or 100 sibling pairs to resemble the positive family history stratum. A binary risk factor with 10% within-sibling correlation was specified at a frequency of 0.5, which closely matches the exposure frequencies of childhood and adulthood direct pesticide application, well water consumption, and farming residences/occupations. Data sets at varying relative risks (RRs) were simulated to determine the minimum detectable RR for 80% power. Statistical power of GEE was based on OR estimates from 1,000 replicates of each simulated data set. The minimum detectable RR was 1.9 when assessing risk factors in 300 sibling pairs, 2.1 in 200 sibling pairs, and 3.0 in 100 sibling pairs.

## Results

Associations of direct pesticide application defined broadly and specifically, well-water consumption, and farming residences and occupations with PD were evaluated in 319 cases from 308 families and 296 relative and other controls. Of the controls, 252 were relative controls from the 308 families with at least one case, and these relative controls included 237 siblings, 10 parents or children, and 5 cousin or avuncular relatives (uncles/aunts/nephews/nieces). The remaining 44 controls were ascertained as spouse or other unrelated controls or as relative controls in families with no environmental risk factor data available on the case(s). Overall, males represented 67.7% of cases and 43.2% of controls. AAE of cases ranged from 29 to 94 years with a mean of 65.6 ± 10.1 years. AAE of controls ranged from 24 to 95 years with a mean of 63.8 ± 12.0 years. Cases were thus more likely to be male (*p *< 0.0001) and older at examination (*p *= 0.0013) when compared to controls. Given these significant differences, sex and AAE were included as confounders in all models. Also, cases reported onset ages ranging from 16 to 85 years with a mean of 57.6 ± 11.5 years and a mean disease duration of 8.0 ± 6.0 years. Only 39 of the 319 cases (12%) reported having symptoms of PD for two years or less.

### Direct pesticide application

Results from models examining associations of childhood and adulthood direct pesticide application with PD, while adjusting for sex, AAE, smoking, and caffeine in the overall data are presented in Table 1. Individuals with PD were 1.61 times as likely to report ever being exposed to pesticides through direct application as their unaffected relatives. Use of protective gear during application did not alter the significant association between history of direct pesticide application and PD (data not shown). The highest exposure categories of frequency, duration, and cumulative exposure and the lowest duration category were also positively associated with PD, and increasing frequency, duration, and cumulative exposure were associated with PD in a dose-dependent manner. These patterns were unchanged when examining adulthood applications only (data not shown).

**Table 1:** Associations of childhood and adulthood direct pesticide application with PD.

	% Exposed*				
				
	**Cases, n = 319**	**Controls, n = 296**	*p *for trend (GEE model 1)**	OR and 95% CI (GEE model 2)**	
History of pesticide use					
Ever	62.7	49.7		1.61	1.13–2.29
Never	37.3	50.3		1.00	Referent
Frequency (days/year)			0.0017		
>10	23.5	13.5		2.07	1.26–3.42
4–10	16.9	13.5		1.41	0.82–2.41
<4	18.8	18.9		1.36	0.85–2.18
Never	37.3	50.3		1.00	Referent
Duration (years)			0.013		
>26	21.9	12.8		1.87	1.16–3.00
11–26	18.2	18.9		1.35	0.84–2.19
<11	18.2	14.2		1.68	1.01–2.80
Never	37.3	50.3		1.00	Referent
Cumulative exposure (days)			0.0023		
>215	21.6	12.2		2.37	1.42–3.94
45–215	18.2	16.2		1.28	0.78–2.11
<45	15.4	14.5		1.43	0.84–2.42
Never	37.3	50.3		1.00	Referent

*Column totals may not sum to 100 due to rounding or missing data.

**GEE model adjusted for AAE, sex, cigarette smoking, and caffeine consumption.

Associations of childhood and adulthood direct pesticide application were also examined in males and females, separately. Significant associations between direct pesticide application and PD were present in both males and females, despite a smaller stratum size and lower application levels for females. A significant positive association between the highest frequency category and PD appeared in both sexes (OR = 2.15; 95% CI: 1.06, 4.35 for males and OR = 2.43; 95% CI: 1.18, 5.01 for females), and a dose-response trend existed in females (*p *= 0.0058). In males, PD was significantly associated with the highest duration (OR = 2.70; 95% CI: 1.35, 5.40) and cumulative exposure (OR = 2.34; 95% CI: 1.14, 4.79) categories, and significant dose-response trends were detected (*p *= 0.021 for duration and *p *= 0.036 for cumulative exposure). In females, PD was significantly associated with the lowest duration category (OR = 2.47; 95% CI: 1.12, 5.44).

Data were then stratified by family history to determine if the associations of childhood and adulthood direct pesticide application differed in individuals with and without a family history of PD. These results are shown in Tables 2 and 3. In the negative family history stratum, all patterns of association resemble those in the overall data (Table 1) with all measures of association being stronger. Ever being exposed, being exposed at the highest exposure categories of frequency, duration, and cumulative exposure, and being exposed at the lowest duration category were all significantly associated with PD. In fact, individuals with PD and no family history were over three times as likely to report being exposed at the highest cumulative exposure level as their unaffected relatives. Increasing frequency, duration, and cumulative exposure were all associated with PD in a dose-response pattern. In contrast, there were no significant associations with PD or significant trends in OR estimates in the positive family history stratum. This stratum, however, represented a smaller portion of the overall data with lower power to detect these size effects (OR < 3) when compared to the negative family history stratum.

**Table 2:** Associations of childhood and adulthood direct pesticide application with PD in negative history families.

	% Exposed*				
				
	**Cases, n = 228**	**Controls, n = 215**	*p *for trend (GEE model 1)**	OR and 95% CI (GEE model 2)**	
History of pesticide use					
Ever	62.7	47.4		1.80	1.20–2.70
Never	37.3	52.6		1.00	Referent
Frequency (days/year)			<0.001		
>10	24.1	11.2		2.55	1.38–4.73
4–10	15.8	13.0		1.58	0.84–2.99
<4	19.7	18.6		1.52	0.89–2.62
Never	37.3	52.6		1.00	Referent
Duration (years)			0.012		
>25	21.9	13.5		1.98	1.14–3.46
10–25	18.4	18.6		1.32	0.76–2.28
<10	17.5	11.6		2.04	1.08–3.88
Never	37.3	52.6		1.00	Referent
Cumulative exposure (days)			<0.001		
>179	21.9	10.7		3.25	1.84–5.73
42–179	18.0	15.3		1.46	0.80–2.68
<42	15.4	14.0		1.48	0.83–2.66
Never	37.3	52.6		1.00	Referent

*Column totals may not sum to 100 due to rounding or missing data.

**GEE model adjusted for AAE, sex, cigarette smoking, and caffeine consumption.

**Table 3:** Associations of childhood and adulthood direct pesticide application with PD in positive history families.

	% Exposed*				
				
	**Cases, n = 91**	**Controls, n = 81**	*p *for trend (GEE model 1)**	OR and 95% CI (GEE model 2)**	
History of pesticide use					
Ever	62.6	55.6		1.20	0.58–2.50
Never	37.4	44.4		1.00	Referent
Frequency (days/year)			0.72		
>11	20.9	19.8		1.36	0.49–3.78
4–11	20.9	14.8		0.96	0.34–2.69
<4	16.5	19.8		1.07	0.40–2.89
Never	37.4	44.4		1.00	Referent
Duration (years)			0.54		
>27	24.2	14.8		1.87	0.79–4.46
13–27	17.6	19.8		1.03	0.39–2.74
<13	17.6	17.3		1.33	0.44–4.04
Never	37.4	44.4		1.00	Referent
Cumulative exposure (days)			0.72		
>242	22.0	13.6		1.21	0.44–3.36
60–242	17.6	19.8		0.88	0.33–2.34
<60	15.4	17.3		1.34	0.44–4.08
Never	37.4	44.4		1.00	Referent

*Column totals may not sum to 100 due to rounding or missing data.

**GEE model adjusted for AAE, sex, cigarette smoking, and caffeine consumption.

### Direct pesticide application of specific functional types and chemical classes

The chemical or trade names of pesticides reported by individuals in our sample were categorized into chemical classes used primarily as herbicides (2,6-dinitroaniline, chlorophenoxy acid/ester, phosphonoglycine, pyridinecarboxylic acid, and triazine), insecticides (alkyl phthalate, botanical, halogenated organic, N-methyl carbamate, microbial, organochlorine, and organophosphorus), and fungicides (azole, guanidine, and thiophthalimide) along with the inorganic/metal class with multiple uses. There were too few individuals with reported applications of the 2,6-dinitroaniline, pyridinecarboxylic acid, alkyl phthalate, halogenated organic, microbial, azole, guanidine, and thiophthalimide classes and too few reports of fungicides to examine these associations with PD. The pesticide names provided by 57 of 200 cases and 54 of 147 controls who reported applying pesticides could not be classified due to either an inadequate report, an unknown trade name, or multiple active chemicals of different classes contained in the reported pesticide.

When examining associations of direct herbicide application with PD, there were significant positive associations between direct pesticide application and PD for both individuals who reported ever applying herbicides (OR = 1.59; 95% CI: 1.00, 2.54) and for individuals who reported ever applying pesticides other than herbicides (OR = 1.61; 95% CI: 1.09, 2.38) as compared to individuals who never applied pesticides. When examining associations between direct insecticide application and PD, only individuals who reported ever applying insecticides were significantly more likely to develop PD (OR = 1.83; 95% CI: 1.20, 2.81). Individuals who reported ever applying pesticides other than insecticides were not significantly associated with an increased risk for PD.

Associations between childhood and adulthood direct pesticide application and PD were then examined for each chemical class by comparing individuals who reported ever applying pesticides from the relevant chemical class and those who reported ever applying other pesticides to the referent group of those who never applied pesticides. These results are summarized in Table 4. For each chemical class, there was a significant positive association of reporting a direct application of other pesticides. However, application of only the organochlorine and organophosphorus chemical classes were found to also be significantly associated with PD. In our sample, chlordane and dichloro-diphenyl-trichloroethane (DDT) were the most common of the 10 organochlorine chemicals, while chlorpyrifos, diazinon, and malathion were the most common of the eight organophosphorus chemicals. Two less common classes, the botanical class (including rotenone) and the chlorophenoxy acid/ester class [including 2,4-dichlorophenoxyacetic acid (2,4-D) and Agent Orange], showed strong OR estimates possibly indicative of a positive association with PD, but these associations were not significant.

**Table 4:** Associations of childhood and adulthood direct pesticide application with PD as divided into chemical classes.

		% Exposed*			
				
**Functional type**	**History of pesticide use for each chemical class**	**Cases, n = 319**	**Controls, n = 296**	OR and 95% CI (GEE model 2)**	
Insecticide	Botanical				
	Ever	2.2	0.3	5.93	0.63–56.10
	Ever to any other pesticide	60.5	49.3	1.57	1.10–2.24
	Never	37.3	50.3	1.00	Referent
	N-Methyl carbamate				
	Ever	11.9	10.8	1.31	0.75–2.28
	Ever to any other pesticide	50.8	38.9	1.70	1.17–2.47
	Never	37.3	50.3	1.00	Referent
	Organochlorine				
	Ever	13.2	7.1	1.99	1.09–3.64
	Ever to any other pesticide	49.5	42.6	1.54	1.07–2.23
	Never	37.3	50.3	1.00	Referent
	Organophosphorus				
	Ever	16.6	10.1	1.89	1.11–3.25
	Ever to any other pesticide	46.1	39.5	1.53	1.05–2.23
	Never	37.3	50.3	1.00	Referent
Herbicide	Chlorophenoxy acid/ester				
	Ever	4.7	2.7	2.07	0.69–6.23
	Ever to any other pesticide	58.0	47.0	1.58	1.11–2.27
	Never	37.3	50.3	1.00	Referent
	Phosphonoglycine				
	Ever	17.9	13.5	1.53	0.92–2.53
	Ever to any other pesticide	44.8	36.1	1.64	1.12–2.40
	Never	37.3	50.3	1.00	Referent
	Triazine				
	Ever	1.6	2.4	1.08	0.32–3.59
	Ever to any other pesticide	61.1	47.3	1.63	1.14–2.33
	Never	37.3	50.3	1.00	Referent
Multiple uses	Inorganic/metal				
	Ever	5.0	3.0	1.51	0.62–3.71
	Ever to any other pesticide	57.7	46.6	1.61	1.13–2.31
	Never	37.3	50.3	1.00	Referent

*Column totals may not sum to 100 due to rounding or missing data.

**GEE model adjusted for AAE, sex, cigarette smoking, and caffeine consumption.

### Well-water consumption

Well-water consumption at childhood and adulthood residences was not significantly associated with PD in our data. Neither a history of well-water consumption nor duration at any exposure level was significantly associated with PD (Table 5). There was also no significant trend in OR estimates for duration of well-water consumption (Table 5). Examination of well-water associations at adulthood residences only did not alter the significance of these results, although the OR estimates were higher than those observed with all residences considered for history (OR = 1.22; 95% CI: 0.87, 1.72) and duration (OR = 1.51; 95% CI: 0.90, 2.56 for >21 years, OR = 1.20; 95% CI: 0.71, 2.03 for 6–21 years, and OR = 1.27; 95% CI: 0.80, 2.02 for <6 years).

**Table 5:** Associations of well-water consumption at childhood and adulthood residences with PD.

	% Exposed*				
				
	**Cases, n = 319**	**Controls, n = 296**	*p *for trend (GEE model 1)**	OR and 95% CI (GEE model 2)**	
History of well water use					
Ever	61.8	59.1		1.08	0.77–1.50
Never	37.6	39.9		1.00	Referent
Duration (years)			0.52		
>22	23.5	20.3		1.17	0.74–1.86
10.5–22	20.1	19.3		1.10	0.71–1.71
<10.5	18.2	18.9		1.02	0.64–1.61
Never	37.6	39.9		1.00	Referent

*Column totals may not sum to 100 due to rounding or missing data.

**GEE model adjusted for AAE, sex, cigarette smoking, and caffeine consumption.

### Farming residences and occupations

Living or working on a farm was not significantly associated with an increased risk of PD in our data as shown in Table 6. Individuals with PD were neither more nor less likely than their unaffected relatives to report ever living or working on a farm or to report any duration level. There was also no significant dose-response relationship between farming residences/occupations and PD. These patterns were unchanged when examining only adulthood farming residences and occupations, and estimated ORs were only slightly higher than those observed with all farming residences and occupations considered (data not shown). There were also no significant associations with PD when examining farming residences and occupations separately (data not shown).

**Table 6:** Associations of childhood and adulthood farming residences and occupations with PD.

	% Exposed*				
				
	**Cases, n = 319**	**Controls, n = 296**	*p *for trend (GEE model 1)**	OR and 95% CI (GEE model 2)**	
History of farming					
Ever	50.8	47.0		1.11	0.80–1.54
Never	47.6	51.4		1.00	Referent
Duration (years)			0.38		
>26	17.9	15.2		1.25	0.80–1.96
16–26	16.9	16.2		1.14	0.73–1.78
<16	16.0	15.2		1.07	0.64–1.77
Never	47.6	51.4		1.00	Referent

*Column totals may not sum to 100 due to rounding or missing data.

**GEE model adjusted for AAE, sex, cigarette smoking, and caffeine consumption.

## Discussion

Our confirmatory findings using a different study design add support to the hypothesis that pesticide exposure is positively associated with risk of PD. Unlike previous studies, our confirmation suggests that this positive association is not likely confounded by unmeasured familial influences, since cases are well-matched to relative controls on genetic and environmental risk factors that may influence disease as well as pesticide and other exposure behaviors. Associations between pesticides and PD were absent in positive history families but were highly significant in negative history families, in which no close relatives of the proband were reported to have PD. The negative history families contain individuals who likely harbor genetic variants that are not sufficient on their own to cause disease but increase the susceptibility for disease development. An environmental insult, such as pesticide exposure, might exacerbate the effect of these genetic susceptibility factors and ultimately lead to sporadic PD. This emphasizes the need to consider pesticides as an important effect modifier in future candidate gene studies. The positive history families contain individuals who may have a stronger genetic predisposition to PD, but the possibility of pesticides and subsequent genetic interactions influencing risk of familial PD cannot be eliminated since our study had less power to detect a pesticides effect in the smaller positive family history stratum. Further, the associations between direct pesticide application and PD were not modified by sex as previously reported [4], but uncertainty of a sex-specific pesticide effect (or lack thereof) remains as other studies have suggested a male-only effect [3,5,6].

Pesticides are a large and diverse group of chemicals, only some of which might be associated with an increased risk of PD. A broad assessment of pesticide exposure likely leads to misclassification and minimizes the magnitude of any observed association, and this likely contributes to the inconsistent findings of an association between broadly defined pesticide exposure and PD [22]. We detected an association of broadly defined pesticides and PD, but our data also implicated specific functional types and chemical classes as associated with an increased PD risk. Similar to previous studies [23-25], both herbicide and insecticide use were shown to have significant positive associations with PD, although the association was stronger for insecticide use. Pesticide exposure was further classified into specific chemical classes, and two insecticide classes (the organochlorine and organophosphorus classes) were shown to significantly increase PD risk. These specific chemical classes have previously been reported to significantly increase the risk of PD [26]. Organochlorines, consisting mostly of chlordane and DDT in our data, have been biologically linked to PD, as brains from PD patients were found to contain higher levels of organochlorine chemicals (notably dieldrin) than brains from control subjects [27,28]. On the other hand, a link between the organophosphorus class and PD is mostly supported clinically with three case studies presenting patients with possible organophosphorus-induced acute parkinsonism [29-31]. We also detected strong associations with two additional classes, the herbicide class of chlorophenoxy acids/esters and the insecticide class of botanticals, although their associations with PD were not significant. Epidemiologic evidence has previously implicated the chlorophenoxy acid/ester class in PD [25], while biological evidence has implicated a botanical chemical (rotenone) in PD with rotenone-injected rodents displaying parkinsonism-like symptoms and pathological features [32,33]. To date, there has been limited data on associations of specific pesticides on PD risk. Our findings show a significant association between broadly defined pesticides and PD that has been narrowed to a few specific chemical classes with insecticide and herbicide functions that warrant further investigation.

Consuming well-water and living or working on a farm have been studied as risk factors for PD, but no previous studies have been able to separate these lifestyle factors as risk factors independent from their correlations with pesticide exposure [34]. Further, meta-analysis of studies conducted in the United States showed that associations for consuming well-water (combined OR = 1.44; 95% CI: 0.92, 2.24) and for living or working on a farm (combined OR = 1.72; 95% CI: 1.20, 2.46) were weaker than associations for direct pesticide application (combined OR = 2.16; 95% CI: 1.95, 2.39) [11]. In our sample, neither consuming well-water nor living/working on a farm were significantly associated with PD. Our sample appears to be representative of a more rural lifestyle and extensive use of pesticides given that few case-control studies conducted in the United States or internationally have reported as high exposure frequencies for the lifestyle factors associated with rural living or pesticide application [2,35]. However, family-based case-control studies using cases and unaffected relatives as controls offer less power than population-based case-control studies to detect effects due to overmatching on exposure history [36-38]. Our sample consists mostly of sibling pairs, who are indeed overmatched on residence during childhood. Given this, we examined associations for well-water consumption and farming residences/occupations when considering adulthood exposures only, but no significant patterns emerged. Relatives, especially siblings, likely continue to be overmatched on residence throughout adulthood. This residual overmatching is a possible explanation for the lack of significant evidence for associations of well-water consumption and farming residences/occupations with PD.

Our results are consistent with the replicated positive association of direct pesticide application with PD [11], but case-control studies are subject to inherent biases. Since environmental exposure data collection occurs retrospectively, recall bias may exist if there was differential accuracy in the information provided by cases and their unaffected relatives. Cases are generally more aware of potential risk factors for disease when compared to population controls thus creating the potential for bias toward positive associations, but the family-based nature of our study might reduce recall bias as unaffected relatives may also be familiar with potential risk factors for disease. Bias in control selection could also impact our study as relative controls who are sampled and those who are not could report differential exposures, particularly if the decision to participate is correlated with closeness to affected relatives and potentially shared exposures. However, family-based controls are typically more motivated than population-based controls to participate, leading to higher participation rates and less bias in control selection. Further, the referral-based ascertainment of prevalent cases and their relatives means that these cases may not be representative of the general population of PD cases and that this study design may not be completely generalizable. It is possible that systematic differences in survival between sampled prevalent and unsampled incident cases are responsible for the apparent association of pesticide exposure and PD. Since our sample consists mostly of cases with longer duration, prevalent case bias could not be controlled by restricting analyses to cases with shorter duration of disease while maintaining sufficient statistical power. And, while estimates of association from samples of unrelated cases and controls from a well-defined population can be validly extrapolated to estimate population risk, this is not true for family-based studies. Nonetheless, we presented strong associations between direct pesticide application and PD in a family-based sample that afforded us the opportunity to reduce confounding by unmeasured influences on exposure and to identify a subset of cases in which the pesticides effect may be enriched. Consistency of our results with many other studies, including prospective cohort studies not subject to such biases [4,5], suggests that these potential biases did not greatly impact our results.

Epidemiologic evidence has mostly supported exposure to pesticides as a risk factor for PD, but biological evidence is presently insufficient to conclude that pesticide exposure causes PD [34]. One proposed mechanism suggests that pesticides cause PD through mitochondrial inhibition as some pesticides are similar to a known parkinsonism-inducing agent, 1-methyl-4-phenyl-1,2,3,6-tetrahydropyridine (MPTP), which exerts its effects by impairing mitochondrial complex I activity [39]. Alternatively, pesticides may exacerbate neuroinflammation by acting as an initiating factor or reacting with mediators of the inflammatory cascade [40]. Although it remains possible that pesticides may be a confounding variable correlated with a truly causal factor for PD [39], many biologically plausible hypotheses exist with most emphasizing the importance of gene-environment interactions in unraveling the complexity of PD development.

## Conclusion

Many epidemiologic studies have examined the relationship between PD and pesticides, but most have been conducted in population-based studies using a broad definition of pesticide exposure. Our findings make three main contributions to the literature supporting pesticide exposure as an important risk factor for PD. First, replication of associations between pesticides and PD in the most extensive family-based study to date suggests that this positive association is not likely confounded by unmeasured genetic and environmental influences on exposure and disease. Secondly, the strongest associations between PD and pesticides were obtained in families with no history of PD thus introducing family history as a potentially important variable to consider in future studies of the effect of pesticides on PD. This finding suggests that sporadic PD cases may be particularly vulnerable to the toxic effects of pesticides, but the possibility of pesticides influencing risk of PD in individuals from families with a history of PD cannot be ruled out. Lastly, our findings add support to the limited data implicating specific classes of pesticides, notably organochlorines, organophosphorus compounds, chlorophenoxy acids/esters, and botanicals, as potential risk factors for PD. Further investigation of these specific pesticides and others may lead to identification of pertinent biological pathways influencing PD development.

## Abbreviations

2,4-D, 2,4-dichlorophenoxyacetic acid; AAE, age-at-examination; CI, confidence interval; DDT, dichloro-diphenyl-trichloroethane; GEE, generalized estimating equations; MPTP, 1-methyl-4-phenyl-1,2,3,6-tetrahydropyridine; OR, odds ratio; PD, Parkinson's disease; RR, relative risk; UPDRS, Unified Parkinson's Disease Rating Scale.

## Competing interests

The author(s) declare that they have no competing interests.

## Authors' contributions

DBH obtained funding, acquired data, performed statistical analyses, analyzed and interpreted data, and drafted and critically revised the manuscript. ERM participated in the study concept and design, obtained funding, supervised the study, analyzed and interpreted data, and critically revised the manuscript. GMM performed statistical analyses and critically revised the manuscript for important intellectual content. JMS, RJ, MAS, and BLS acquired data and critically revised the manuscript for important intellectual content. JMV participated in the study concept and design, obtained funding, supervised the study, and critically revised the manuscript for important intellectual content. WKS participated in the study concept and design, obtained funding, supervised the study, analyzed and interpreted data, and critically revised the manuscript for important intellectual content. All authors read and approved the final manuscript.

## Pre-publication history

The pre-publication history for this paper can be accessed here:



## Supplementary Material

Additional file 1 Risk factor questions used to assess residences, occupations, and pesticide applications. The portions of our PD risk factor questionnaire that pertain to this work, including residence history, occupational history, and pesticide applications, are presented. Click here for file
