# Supplementary material for: Pesticide exposure and risk of Parkinson's disease: A family-based case-control study
Source: BMC Neurol. 2008 Mar 28;8:6. doi: 10.1186/1471-2377-8-6 (PMC2323015; doi:10.1186/1471-2377-8-6)
Supplement: Additional file 1 — Risk factor questions used to assess residences, occupations, and pesticide applications. The portions of our PD risk factor questionnaire that pertain to this work, including residence history, occupational history, and pesticide applications, are presented. [file 1471-2377-8-6-S1.pdf]

3. ***I would like you to list the cities, towns, or communities in which you have lived for the majority of each year from childhood to now. For example, if you were away at school for most of the year but returned home for summer vacation, please list your school as your residence for that time period. I will ask several questions about each place you name. Let's begin with your childhood home (Residence #1) - what was the name of the place you were born?***

[Interviewer: GO TO the "PD Risk Factor Questionnaire: Residence History" Form. Complete as many forms as necessary to record all residences, then enter the total number of residences below.]

Record the total number of RESIDENCES here: ☐☐

4. Other than the places you just named, have you ever lived on or next to a farm for over a month but less than six months? ☐ Yes ☐ No ☐ Don't know

If NO or Don't Know, skip to Question 5
If YES:

a) When was the first time you lived on or next to a farm for more than a month but less than six months? Year: ☐☐☐☐ (or AGE: ☐☐)

b) When was the last time you lived on or next to a farm for more than a month but less than six months? Year: ☐☐☐☐ (or AGE: ☐☐)

c) About how many months overall did you live on or next to farms for more than a month but less than six months at a time? ☐☐☐ ☐ Don't Know

d) In any of the years you lived there, did you drink well water? ☐ Yes ☐ No ☐ Don't know

IF YES: While living on or next to these farms, how many months overall did you drink well water? ☐☐☐ ☐ Don't Know

e) What was raised on these farms? ☐ Crops ☐ Livestock ☐ Both ☐ Don't know

5. ***I would like to discuss the full or part-time jobs that you have held for a year or longer. For each job, I will ask you for the job title, the company name, how long you worked there, and your major duties. Let's begin with your first job.***

[Interviewer: GO TO the "PD Risk Factor Questionnaire: Occupation History" Form. Complete as many forms as necessary to record all jobs, then enter the total number of jobs below. CODE THE JOB TITLE AFTER THE INTERVIEW.]

Record the total number of JOBS here: ☐☐

PD Risk Factor Questionnaire: RESIDENCE History

Center: **Family#:** **Individual#:** **Individual's name:** **Date of Birth:**

Day / Month* / Year

Exam Date: Day / Month* / Year

Examiner:

RESIDENCE # ______ *(Sequence Number)*

Name of City, Town, or Community:

What year(s) did you live there? (**OR AGE**) ○ Don't know

About how many people lived in that city, town, or community? ○ Don't know

In any of the years you lived there, did you drink well water? ○ Yes ○ No ○ Don't know

If YES: For which years did you drink well water?

(**OR AGE**) ○ Don't know

Did you live on or next to a farm? ○ Yes ○ No ○ Don't know

If YES: What was raised there? ○ Crops ○ Livestock ○ Both ○ Don't know

RESIDENCE # ______ *(Sequence Number)*

Name of City, Town, or Community:

What year(s) did you live there? (**OR AGE**) ○ Don't know

About how many people lived in that city, town, or community? ○ Don't know

In any of the years you lived there, did you drink well water? ○ Yes ○ No ○ Don't know

If YES: For which years did you drink well water?

(**OR AGE**) ○ Don't know

Did you live on or next to a farm? ○ Yes ○ No ○ Don't know

If YES: What was raised there? ○ Crops ○ Livestock ○ Both ○ Don't know

RESIDENCE # ______ *(Sequence Number)*

Name of City, Town, or Community:

What year(s) did you live there? (**OR AGE**) ○ Don't know

About how many people lived in that city, town, or community? ○ Don't know

In any of the years you lived there, did you drink well water? ○ Yes ○ No ○ Don't know

If YES: For which years did you drink well water?

(**OR AGE**) ○ Don't know

Did you live on or next to a farm? ○ Yes ○ No ○ Don't know

If YES: What was raised there? ○ Crops ○ Livestock ○ Both ○ Don't know

***Valid month entries are: JAN,FEB,MAR,APR,MAY,JUN,JUL,AUG,SEP,OCT,NOV,DEC**

PD Risk Factor Questionnaire: OCCUPATION History

Center: ☐☐☐ **Family#:** ☐☐☐☐☐ **Individual#:** ☐☐☐☐ **Individual's name:** ________________ **Date of Birth:** ☐☐ / ☐☐☐ / ☐☐☐☐
Day Month* Year

Exam Date: ☐☐ / ☐☐☐ / ☐☐☐☐ **Examiner:** ☐☐☐☐☐☐☐☐☐☐☐☐☐☐☐☐☐
Day Month* Year

JOB # ______ *(Sequence Number)* **JOB CODE:** ☐☐☐☐☐☐

Job Title:

Company Name:

What year(s) did you work there? ☐☐☐☐ ☐☐☐☐ **(OR AGE** ☐☐ ☐☐ **)** ○ Don't Know

How many hours a week? ☐☐☐ **How many weeks per year?** ☐☐

What were your major duties?______________________________

JOB # ______ *(Sequence Number)* **JOB CODE:** ☐☐☐☐☐☐

Job Title:

Company Name:

What year(s) did you work there? ☐☐☐☐ ☐☐☐☐ **(OR AGE** ☐☐ ☐☐ **)** ○ Don't Know

How many hours a week? ☐☐☐ **How many weeks per year?** ☐☐

What were your major duties?______________________________

JOB # ______ *(Sequence Number)* **JOB CODE:** ☐☐☐☐☐☐

Job Title:

Company Name:

What year(s) did you work there? ☐☐☐☐ ☐☐☐☐ **(OR AGE** ☐☐ ☐☐ **)** ○ Don't Know

How many hours a week? ☐☐☐ **How many weeks per year?** ☐☐

What were your major duties? ______________________________

***Valid month entries are: JAN, FEB, MAR, APR, MAY, JUN, JUL, AUG, SEP, OCT, NOV, DEC**

Now I would like to ask you a few questions about substances you may have worked with or been exposed to at work or home.

# 1. Have you ever applied pesticides to kill weeds, insects, or fungus at work, in your home, in your garden, or on your lawn? ○ Yes ○ No ○ Don't know

[INTERVIEWER: If the participant answered NO or Don't know to question # 1 please skip to question # 3.]

# 2. Please tell me the name of any pesticide you can remember using. I will ask several questions about each pesticide you name.

*[INTERVIEWER: **GO TO** "PD Risk Factor Questionnaire: Pesticide History" Form. Complete as many forms as necessary to record all pesticides, then enter the total number of pesticides, then enter the total number of pesticides below.]*

Record the total number of PESTICIDES here: ☐☐

PD Risk Factor Questionnaire: PESTICIDE History

Center: ☐☐☐ **Family#:** ☐☐☐☐☐ **Individual#:** ☐☐☐☐ **Individual's name:** ________ **Date of Birth:** ☐☐ / ☐☐☐ / ☐☐☐☐ (Day / Month* / Year)

Exam Date: ☐☐ / ☐☐☐ / ☐☐☐☐ (Day / Month* / Year) **Examiner:** ☐☐☐☐☐☐☐☐☐☐☐☐☐☐☐☐☐☐

PESTICIDE #_____ *(Sequence Number)*

1) Name of the pesticide:

a) In what year did you begin? Year: ☐☐☐☐ (or AGE: ☐☐)

b) Have you stopped? ○ Yes ○ No ○ Don't Know

If **YES:** What year did you stop? Year: ☐☐☐☐ (or AGE: ☐☐)

c) How many days per year did you use these pesticides? ☐☐☐ ○ Don't know

d) Did you usually use protective gear, such as a mask, rubber gloves, or rubber boots? ○ Yes ○ No ○ Don't know

e) How did you apply the pesticide?
- ○ Spraying by hand
- ○ Spraying by tractor
- ○ Spraying by airplane
- ○ Spreading solid granules
- ○ Putting in irrigation water
- ○ Placing pest strips or traps
- ○ Other

PESTICIDE # _____ *(Sequence Number)*

2) Name of the pesticide:

a) In what year did you begin? Year: ☐☐☐☐ (or AGE: ☐☐)

b) Have you stopped? ○ Yes ○ No ○ Don't Know

If **YES:** What year did you stop? Year: ☐☐☐☐ (or AGE: ☐☐)

c) How many days per year did you use these pesticides? ☐☐☐ ○ Don't know

d) Did you usually use protective gear, such as a mask, rubber gloves, or rubber boots? ○ Yes ○ No ○ Don't know

e) How did you apply the pesticide?
- ○ Spraying by hand
- ○ Spraying by tractor
- ○ Spraying by airplane
- ○ Spreading solid granules
- ○ Putting in irrigation water
- ○ Placing pest strips or traps
- ○ Other

***Valid month entries are: JAN, FEB, MAR, APR, MAY, JUN, JUL, AUG, SEP, OCT, NOV, DEC**
